# Ability of the integrated pulmonary index to predict impending respiratory events in the early postoperative period

**DOI:** 10.1186/s13741-023-00322-2

**Published:** 2023-07-17

**Authors:** Stephen Probst, Jamie Romeiser, Tong J. Gan, Darcy Halper, Andrew R. Sisti, Hiroshi Morimatsu, Kentaro Sugimoto, Elliott Bennett-Guerrero

**Affiliations:** 1 Department of Anesthesiology, Stony Brook University Medical Center, 101 Nicolls Road, Stony Brook, NY 11794 USA; 2 Department of Anesthesiology and Resuscitology, Okayama University Hospital, Okayama, Japan

## Abstract

**Background:**

In the early postoperative period, respiratory compromise is a significant problem. Standard-of-care monitoring includes respiratory rate (RR) and pulse oximetry, which are helpful; however, low SpO_2_ is often a late sign during decompensation. The FDA-approved Capnostream-20p monitor records four variables (SpO_2_, RR, End-tidal CO_2_, heart rate), which are combined by *fuzzy logic* into a single, unit-less value (range 1–10) called the integrated pulmonary index (IPI). No published studies have assessed the performance of a low IPI to predict impending respiratory events.

**Methods:**

In this investigator-initiated study, adult patients undergoing general anesthesia were monitored with the Capnostream-20p monitor for up to 2 h during their recovery room stay. The study coordinator, who along with clinicians, was blinded to IPI values, recorded the time of any respiratory event, defined a priori as any one of eight respiratory-related interventions/conditions. The primary sensitivity endpoint (*early detection success*) was defined as at least 80% of events predicted by at least 2 consecutive low IPI (≤ 7) values within 2–15 min *before* an event occurred. *Late detection* was defined as low IPI values occurring with 2 min prior to or 2 min after the event occurred.

**Discussion:**

Of 358 patients, ≥ 1 respiratory event occurred in 183 (51.1%) patients. Of 802 total events, 606 were detected early (within 2–15 min prior to the event), and 653 were detected either early or late. Therefore, the sensitivity for early detection was 75.6% (95% confidence interval [CI]: 72.6–78.5%), which differed significantly from the 80% sensitivity goal by 4.4% (*p* = 0.0016). Sensitivity for total success (early or late) was 81.4% (95% CI: 78.7–84.1%), which was significantly different from the 90% on time sensitivity goal by 8.6% (*p* < 0.0001).

**Conclusions:**

A low IPI was 75.6% sensitive for *early detection* (within 2–15 min) prior to respiratory events but did not achieve our preset threshold of 80% for success.

**Supplementary Information:**

The online version contains supplementary material available at 10.1186/s13741-023-00322-2.

## Background

In the first few hours after emergence from general anesthesia, respiratory compromise can be a significant problem due to multiple factors including effects of the residual anesthetic agents, atelectasis from postoperative splinting secondary to pain, and opioid-induced respiratory depression (Arozullah et al. 2000; Nagappa et al. 2017; Hedenstierna and Edmark 2010). Postoperative pulmonary complications can significantly increase morbidity, mortality, length of stay, and healthcare costs (Arozullah et al. 2000; Hines et al. 1992; Langeron et al. 2014). The standard of care for monitoring in the early post-operative period includes respiratory rate (RR) and pulse oximetry (SpO_2_), which are helpful; however, a low SpO_2_ can be a late sign during a patient’s deterioration (Lam et al. 2017; Yildirim 2018). There is a need for monitors that can continuously assess and alert clinicians as early as possible to impending respiratory compromise.

The FDA-approved Capnostream 20p monitor (Medtronic, Boulder, USA) has been proposed as a potentially useful tool for this type of early recognition. It records four variables (SpO_2_, RR, non-invasive EtCO_2_, heart rate) every 30 s, which are then integrated by *fuzzy logic* into a single, unit less, value called the integrated pulmonary index (IPI). The IPI can range from 1 to 10 with 4 and under requiring intervention and 8 to 10 representing the normal range (https://www.medtronic.com/covidien/en-us/products/capnography/capnography-patient-education.html). A subset analysis of patients from the PRODIGY study assessed respiratory depression episodes in patients receiving opioids and found that detection of respiratory depression episodes by the monitor correlated with the PRODIGY risk score. This study, however, measured IPI overnight in the general ward (not PACU), and the analysis did not assess real-time relationships between changes in IPI values and whether these predict a respiratory event or treatment soon thereafter. Another study showed that patients with low IPI and SpO_2_ values on admission to the PACU were more likely to develop respiratory compromise during their PACU stay (Kuroe et al. 2021); however, the study did not assess the performance of the monitor to warn them of impending issues. Finally, an extremely low IPI value (= 1) was used in a small randomized trial (vs. standard of care) of patients staying in the hospital overnight to trigger nurse-led respiratory treatments (Broens et al. 2021). They found that patients randomized to monitoring had more nurse-led interventions and a fewer number of respiratory events.

Therefore, while several studies included IPI measurements, no published studies, to our knowledge, have assessed the real-time predictive value of this monitor in patients recovering from general anesthesia. Therefore, we designed a prospective blinded study to test the hypothesis that an abnormal IPI predicts impending respiratory events in the early postoperative period.

## Methods

### Funding and role of sponsor

This study was investigator-initiated and was designed by the investigators. The device’s manufacturer (Medtronic) provided disposable probes, monitors, and funding for investigator/research coordinator effort through their investigator-initiated studies program. Medtronic otherwise played no role in the design, execution, and analysis of data for the study. Furthermore, the manuscript was drafted only by the investigators, and Medtronic did not approve or play any role in the drafting/revision of the manuscript.

### Study design and patient population

This prospective, observational study was conducted following approval by the Stony Brook University Hospital Institutional Review Board (protocol IRB # 1037010). Another study site was planned; however, all data from this site were excluded since it appeared that event data were not captured systematically and in real time by a study team member, as was done at the US site. The power of our study, excluding the other site’s data, was adequate (see Statistical Methods section). This observational study did not require clinical trial registration. Written informed consent was obtained from all participants.

Adult patients (≥ 18 years) undergoing general anesthesia for one of the following procedures at Stony Brook Hospital were included: craniotomy, open upper abdominal surgery (including bariatric), vascular surgery (including open aortic surgery and lower extremity bypass surgery), and surgical repair of hip fracture. Patients undergoing hip fracture surgery had to have at least one of the following risk factors: albumin < 30 g/L, BUN > 30 mg/dL, dependent functional status, COPD, and age > 75. These patients were selected to increase the likelihood of post-operative respiratory events (Attaallah et al. 2019; Gupta et al. 2012).

Subjects who were pregnant, unable to provide consent, or mechanically ventilated prior to surgery were excluded.

### Study procedures

Since this was an observational and blinded study, clinical care was administered per our hospital’s standard of care. Following surgery, patients were transported to the post-anesthesia care unit (PACU) or intensive care unit (ICU) and monitored in a standard manner for that unit. At a minimum, this included continuous heart rate monitoring with ECG, pulse oximetry, respiratory rate, non-invasive or invasive blood pressure, and temperature.

The study personnel attached the Capnostream 20p monitor (Medtronic, USA) when patients arrived in the PACU or ICU. The Capnostream 20p device consists of two components, a disposable nasal cannula and a disposable pulse oximeter probe. This monitor measures the following four respiratory-related variables continuously: respiratory rate, pulse rate, SpO_2_, and end-tidal CO_2_. These disposables were connected to the Capnostream 20p portable monitor, which was located at the patient’s bedside. The monitor records data every 30 s onto the device’s memory. *Fuzzy logic* is used to produce a pulmonary index (IPI) value which is integrated from the 4 measured variables. The IPI is unit less and ranges from 1 to 10 and per the manufacturer values 4 and under require intervention, and values of 8 to 10 represent the normal range (https://www.medtronic.com/covidien/en-us/products/capnography/capnography-patient-education.html). The clock on the monitor was synchronized with the time on the hospital’s electronic medical record (EMR).

The care team and patient were both blinded to the Capnostream 20p monitor’s data at all times by affixing an opaque screen to the monitor’s screen and disabling all visual and audible alarms. The study personnel attached the probes and monitor, and after ensuring that it was working properly and recording all four variables and generating an IPI value, they covered the screen. Therefore, the study personnel were also blinded to IPI values during data collection.

IPI data collection was initiated as soon as the patient arrived in the PACU or ICU, after anesthesia care team handover to the nurse, and when the IPI value was greater than 7. This was done to exclude patients who arrived in respiratory compromise, in which case a “normal” baseline would not exist in order to study whether a low IPI (≤ 7) predicts initiation or escalation of treatment for respiratory issues (primary objective of the study). Patients were allowed up to 60 min to achieve this “normal” baseline, and if not achieved, the patient was deemed a screen failure and not eligible for analysis for the primary objective. Patients whose surgical plan was changed, or who did not undergo general anesthesia, or remained intubated during the first 60 min in the PACU or ICU were also a priori determined to be screen failures.

IPI data were recorded during the patient’s entire PACU/ICU stay or 2 h, whichever was shorter. At the conclusion of monitoring, the study coordinator downloaded the raw data file for analysis by a statistician (co-author JR).

A research coordinator remained at the patient’s bedside for the entire period of data collection. This was done to avoid reliance on the timing of nurse-charted interventions, which, even if off by a few minutes, would impact our study’s validity. The study coordinator recorded the time on the synchronized clock when any of the following interventions were initiated or ordered: (1) increase in oxygen delivery, (2) desaturation defined as SpO_2_ less than 90% for > 2 min, (3) inhaled bronchodilator (i.e., albuterol, ipratropium bromide, or racemic epinephrine), (4) non-invasive (e.g., BiPap) or intubation/mechanical ventilation, (5) naloxone or flumazenil administration, (6) hypercarbia defined as pCO_2_ greater than 50 mm Hg by arterial blood gas analysis, (7) respiratory rate < 8, and (8) epinephrine or atropine intravenously. These events were defined a priori and were chosen since they reflect interventions and/or conditions associated with respiratory compromise or deterioration.

#### Data management and statistical analysis

Data were recorded into a dedicated REDCap software database. Categorical variables are described as frequencies and percentages, and continuous variables are described as either means (standard deviation [SD]) or medians (interquartile range [IQR]). All analyses were performed using the SAS 9.4 © software (Cary, NC).

As described in more detail below, our statistical plan assessed the performance of this device in two major ways: (1) to assess the sensitivity of the device in patients who experienced a respiratory event (primary objective/analysis) and (2) *false discovery rate* (i.e., the proportion of “false alarms” out of the total number of detections), defined as patients with a low IPI value (≤ 7) who did not proceed to have a respiratory event.

Our primary objective was to assess the sensitivity of the device in patients who experienced a respiratory event. The primary hypothesis was that at least 80% of events would be predicted by a low IPI. *Early detection success* was predefined as an IPI value of ≤ 7 sustained for two consecutive 30-s measurements, at any point within 2–15 min before any one of the 8 events defined above was recorded. *Late detection* was predefined as an IPI value of ≤ 7 sustained for two consecutive 30-s measurements, at any point within 0–2 min before or 0–2 min after an event. All recorded events for each patient were assessed and included in the sensitivity analyses. We defined *total success* as both early and late detection. We set an a priori level of 80% for *early detection success* and 90% for *total success*. A one-sample *z* test for proportions was used to determine if the observed sensitivity for early and total success was different from the 80% and 90% goals, respectively, set a priori.

Expected rates and actual rates of success for both early and late detection of events were compared using a one sample *z*-test for proportions. The primary endpoint was sensitivity of early event detection. We anticipated an 80% target success rate for *early detection* success and powered the study to detect a 15% difference below this anticipated success rate (or, 65%). Using a one-sided test procedure, with an alpha of 5%, at 80% power, the number of events needed for a one-sample inference for a binomial proportion was 56. While the anticipated event rate was 10%, our true observed event rate per person was 51.1%, with 183 participants having an event; therefore, the number of participants with events was over three times the amount necessary to adequately power the study.

#### False discovery rates

Low IPI events were identified as a sustained IPI of ≤ 7 for 2 consecutive readings (i.e., 1 min). The first IPI event for each patient was selected, and data examined to determine whether or not an observed event occurred 2–15 min after the reading (i.e., early successful prediction of event). An observed low IPI was deemed a partial success (late detection) if an observed event occurred 2 min before through 2 min after the low reading. Once the 15-min prospective window of observation passed, a washout period of 30 min was applied, and detection for low sustained IPI values continued. Therefore, based on this washout period, participants could have up to 3 low IPI events during the 2-h observation period. False discovery rates were calculated as the number of false positive detections out of the total number of positive detections. We expected the false discovery rate would be no more than 35%.

One observation per subject was selected for a final examination of sensitivity and specificity. The first occurrence of either a respiratory event or a low IPI was selected and evaluated for early or on-time success. Participants with neither an observed respiratory event nor low IPI were marked appropriately as no event/negative test.

## Results

**Fig. 1:**
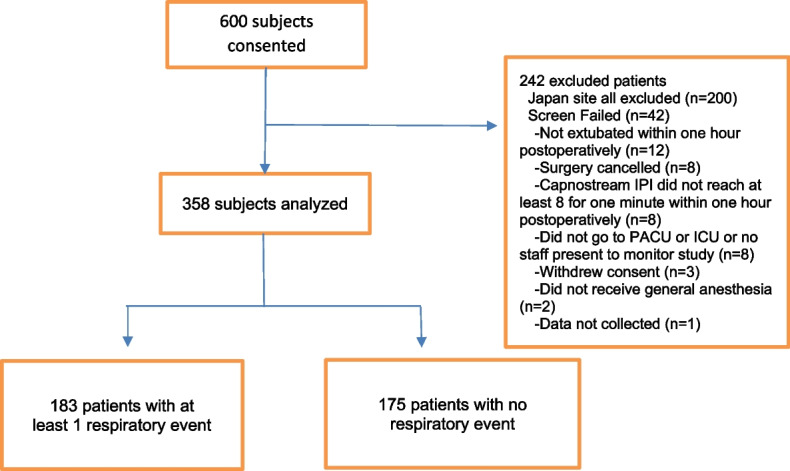
Enrollment

As shown in Fig. 1 (CONSORT diagram), 600 patients were consented between November 2017 and June 2020 at two sites (Stony Brook University, NY, USA, *n* = 400, Okayama University, Japan *n* = 200). Prior to the statistical analyses, all data from the site in Japan were excluded since it appeared that event data were not captured systematically and in real time by a study team member, as was done at the US site. The final number of subjects analyzed was 358.

Preoperative characteristics are shown in Table 1. Intraoperative characteristics are shown in Table 2. Of note, the mean (SD) duration of surgery was 197.2 (104.5) min. The mean (SD) time from entry into the PACU or ICU to initiation of monitoring of the IPI was only 14.3 (10.5) minutes. The IPI was monitored (median, IQR) for 120 (117–120) min). Of the 358 participants, 46 (12.85%) spent less than 2 h in the PACU, and 312 (87.15%) spent ≥ 2 h in the PACU (Table 3).

**Table 1:** Preoperative demographic and clinical characteristics

Total participants	358	
Age (mean, SD)	57.13	14.43
Sex (*n*, %)		
Male	199	55.6%
Female	159	44.4%
Race/ethnicity (*n*, %)		
Black or African American	20	5.6%
White	317	88.5%
Hispanic	28	7.8%
Others	21	5.9%
Respiratory diseases (*n*, %)		
COPD	21	5.9%
Home oxygen use	0	0.0%
Asthma	37	10.3%
Daily inhaled corticosteroids	10	2.8%
Albuterol, PRN, or daily	28	7.8%
Restrictive lung disease	3	0.8%
OSA (*n*, %)	55	15.4%
Stop BANG score recorded (*n*, %)	334	93.3%
Snore	109	32.6%
Tired during day	70	21.0%
Stop breathing while sleeping	46	13.8%
Hypertension	174	52.1%
Total score (*n*, %)		
0	103	30.8%
1	122	36.5%
2	62	18.6%
3–4	47	14.1%
Tobacco Hx (*n*, %)		
Never	159	44.4%
Current	41	11.5%
Previous	158	44.1%
Congestive heart failure (*n*, %)	10	2.8%
Hypertension (*n*, %)	180	50.3%
Diabetes (*n*, %)	52	14.5%
GFR < 60 (*n*, %)	58	16.2%
Chronic opioid use (*n*, %)	42	11.7%
Functional status (*n*, %)^a^		
< 4 METS	2	0.6%
≥ 4 METS	315	88.0%
Limited by pain/disability	41	11.5%

*SD* standard deviation, *COPD* chronic obstructive pulmonary disease, *OSA* obstructive sleep apnea, *GFR* glomerular filtration rate, *METS* metabolic equivalents

^a^Functional status was obtained from preoperative services clinic note

**Table 2:** Perioperative characteristics

ASA class (*n*, %)		
I	11	3.1%
II	104	29.1%
III	231	64.5%
IV	12	3.4%
Surgery duration (min, mean SD)	197.2	104.5
Type of procedure (*n*, %)		
Craniotomy	55	15.4%
Hip fracture repair	4	1.1%
Open upper abdominal	152	42.5%
Vascular	10	2.8%
Posterior spinal fusion	137	38.3%
General anesthesia used (*n*, %)	358	100%
Regional anesthesia used (*n*, %)	14	3.9%
Spinal or epidural	10	2.8%
PNB or catheter	4	1.1%
Intraoperative transfusion (*n*, %)	63	17.6%
Cell saver	38	10.6%
RBC	22	6.1%
FFP	2	0.6%
Platelets	2	0.6%
Autologous blood	3	0.8%
Cryoprecipitate	0	0.0%
Intraoperative fluids		
Crystalloids (median, IQR)	1754	1100, 2700
Albumin (*n* = 61) (mean, SD)	284.43	211.25
Hetastarch (*n* = 5) (mean, SD)	450	111.8
Urine output (mean, SD)	453.91	483.7
Intraoperative medications (*n*, %)		
Opioids (IV)	358	100.0%
Fentanyl	356	99.4%
Morphine	4	1.1%
Hydromorphone	162	45.3%
Remifentanil	55	15.4%
Sufentanil	1	0.3%
Neuromuscular blocker	347	96.9%
Reversal	330	92.2%
Sugammadex	302	84.4%
Neostigmine	29	8.1%
Extubated in operating room (*n*, %)	357	99.7%
ICU admission (*n*, %)	51	14.2%
Discharge ASU or HOME (*n*, %)	45	12.6%
Death/in-hospital mortality within 30 days (*n*, %)	0	0.0%
Postoperative length of stay (mean, SD)	4.03	4.55
Time in PACU (median, IQR)	258	(157, 416)
Time from PACU entry to monitor start (min, mean, SD)	14.3	10.5
Capnostream monitor duration (min, median, IQR)^a^	120	117, 120

*ASA* American Society of Anesthesiologists, *PNB* peripheral nerve block, *RBC* red blood cells, *FFP* fresh frozen plasma, *ICU* intensive care unit, *ASU* ambulatory surgical unit, *PACU* post-anesthesia care unit

^a^Median time monitored was 120 min (IQR 117, 120); 91 (25.4%) patients had less than 120 min of monitoring, but only 22 (6.1%) patients had less than 60 min of monitoring time

**Table 3:** Exploratory: one observation per subject was selected for a final examination of sensitivity and specificity. The first occurrence (in time) of either an event or a low IPI was selected evaluated for early or on time success. Participants with neither an observed event or low IPI were marked appropriately as no event/negative test

	**Corresponding event (observed)**		
**LOW IPI (test)**	Event +	Event −	
Positive	**83**	**188**	
Negative	**44**	**43**	
	**Estimate**	**Lower 95% CI**	**Upper 95% CI**
**Sensitivity**	**65.4%**	57.1%	73.6%
**Specificity**	**18.6%**	13.6%	23.6%
**Negative predictive value**	**49.4%**	25.1%	36.1%
**Positive predictive value**	**30.6%**	38.9%	59.9%

*IPI* integrated pulmonary index, *CI* confidence interval

**Fig. 2:**
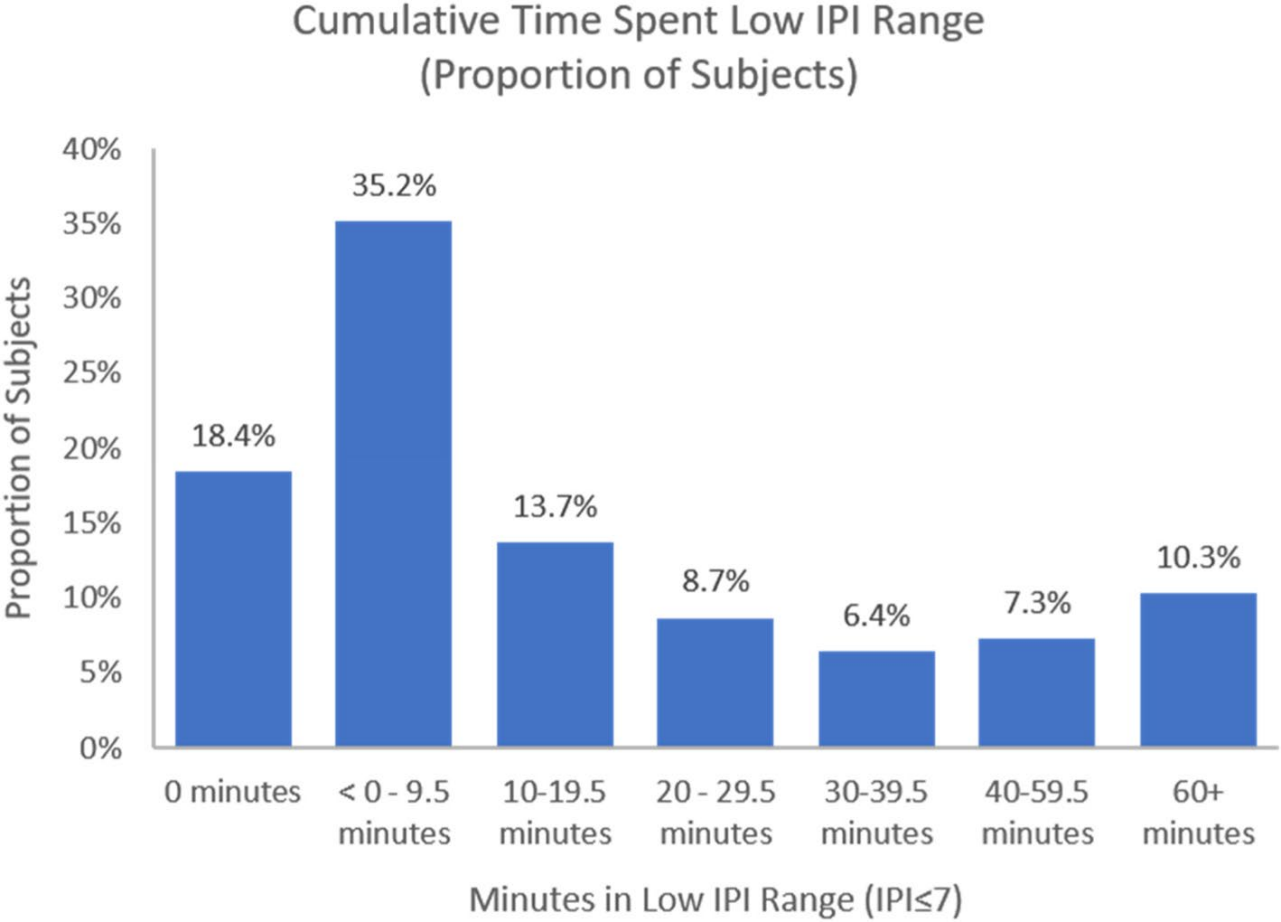
Cumulative time spent low IPI range (proportion of subjects)

All low IPI events were tallied per participant over the monitoring period (Fig. 2). 18.4% (*n* = 66) participants had 0 low IPI events. 35.2% (*n* = 126) of participants had between 1 and 19 low IPI events, which at maximum is roughly equivalent to between 0.5 and 9.5 min of low IPI time (2 IPI events per minute).

### Sensitivity results (primary analysis)

**Fig. 3:**
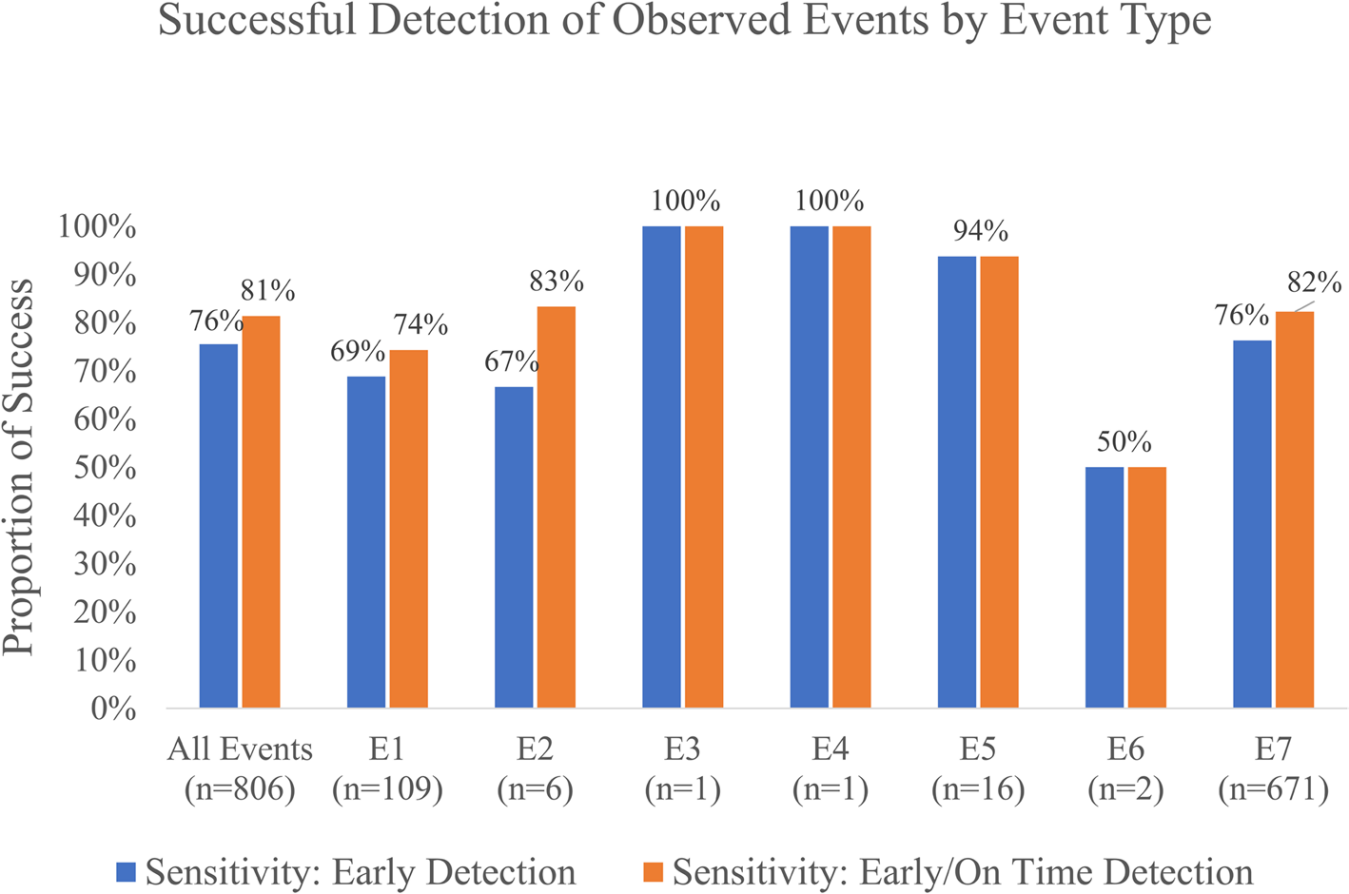
Successful detection of observed events by event type. (E1) Increase in oxygen delivery method (such as nasal cannula to high flow nasal cannula or non-rebreather). (E2) Respiratory treatment (including albuterol, Atrovent, or racemic epinephrine). (E3) Initiation of BiPap or intubation/mechanical ventilation. (E4) Administration of naloxone or flumazenil. (E5) Desaturation—defined as pulse oximetry below 90% for greater than 2 min sustained. (E6) Hypercarbia—defined as PaCO_2_ (from ABG) > 50 mm Hg. (E7) Respiratory rate < 8 bpm. (E8) Administration of atropine or epinephrine at any dose (none observed)

The total number of participants with at least 1 observed event was 183 (51.1%) of 358. There were 802 events total. Of these events, 606 were detected early, and 653 were detected either early or late. Sensitivity for early detection was 75.6% (95% confidence interval [CI]: 72.6%, 78.5%), which differed significantly from the 80% sensitivity goal by 4.4% (*p* = 0.0016). Sensitivity for total success (early or late) was 81.4% (95% CI: 78.7%, 84.1%), which also significantly different from the 90% on time sensitivity goal by 8.6% (*p* < 0.0001). Successful detection of observed events by event type, e.g., desaturation defined as pulse oximetry below 90% for greater than 2 min sustained, is shown in Fig. 3.

### False discovery results

The total number of participants with at least 1 low IPI was 292 (81.6%). There were 586 low IPI events total. Of these events, 145 were early detectors of an observed event, and 173 were either early or late detectors of an observed event. The false discovery rate for early detection was 75.3% (95% confidence interval [CI]: 71.6%, 78.7%), and 70.5% (95% CI: 66.7%, 74.2%) for late or early detection. Both were significantly higher than the 35% goal (both *p* < 0.0001).

Supplemental Table 1 shows an exploratory analysis of false discovery rates for all low IPI events but with no washout period. Supplemental Table 2 shows sensitivity and specificity considering only the first occurrence (in time) of either an event or a low IPI value for early or on time success.

## Discussion

This prospective study sought to determine if there was a temporal relationship between a low IPI and a respiratory event occurring within several minutes in patients recovering from general anesthesia. During the maximum 2-h monitoring period, we observed 802 total respiratory events in 358 patients. A low IPI was 75.6% sensitive for *early detection*, within 2–15 min prior to the respiratory events but did not achieve our preset threshold of 80% for success.

After general anesthesia, respiratory adverse events or complications are a significant problem due to residual anesthetic, atelectasis from surgery, postoperative splinting from pain, and opioid induced respiratory depression. The standard of care of monitoring in most patients includes pulse oximetry, but its decrease is often a late sign. There is a need for better monitors in the post-operative setting that can continuously assess and alert clinicians as early as possible that the patient is beginning to deteriorate. The FDA-approved Capnostream monitor has been proposed as a potentially useful tool for early recognition as it combines 4 commonly used variables, i.e., SpO_2_, RR, EtCO_2_, pulse rate. The utility of this monitor can be assessed in several ways. Our study sought to determine if the monitor could provide early warning of impending events, whereas previous studies had a different focus (Kuroe et al. 2021; Broens et al. 2021; Khanna et al. 2020; Driver et al. 2021).

The PRODIGY study’s primary objective was to develop a risk prediction tool in 1335 patients on the hospital general care floor who were receiving opioids (Khanna et al. 2020). A separate publication presented a post hoc analysis involving a subset of these patients (*n* = 250) in whom IPI values were recorded with the Capnostream monitor (Driver et al. 2021). Their subset analysis found that detection of respiratory depression episodes by the monitor correlated with the PRODIGY risk score (Driver et al. 2021). This analysis differed from our study in two ways. First, in PRODIGY, recording of the IPI began at a later phase of care, i.e., median 4.3 h after the end of surgery, whereas in our study, monitoring of IPI began on average 14 min after arrival in the PACU. In the PRODIGY study, monitoring focused on the time when patients were recovering on the general ward (not recovery room or ICU as in our study) with a median duration of monitoring in their study of 22.3 h. Secondly, their subset analysis focused on general associations over several hours and did not assess if there was a more immediate relationship between a decrease in an IPI value and a subsequent respiratory event.

In contrast to PRODIGY, a study by Kuroe et al. recorded IPI values with the Capnostream monitor closer to the end of surgery (Kuroe et al. 2021). They found, not surprisingly, that patients with low IPI and SpO_2_ values on admission to the PACU were more likely to develop respiratory compromise during their PACU stay (Kuroe et al. 2021). In contrast to our study, this study was not blinded and did not assess the immediate relationship between a decrease in IPI values and a subsequent respiratory event. In another study, Broens et al. used an extremely low IPI value of 1 to trigger nurse led respiratory treatments in a small randomized trial (vs. standard of care) of patients staying in the hospital overnight (Broens et al. 2021). They found that patients randomized to monitoring had more nurse led interventions and a fewer number of respiratory events. Of note, this study was not blinded, did not take place in the PACU, and did not assess the temporal relationship between a low IPI and a respiratory event.

Our study has several limitations. Our results may reflect care in a setting which may be different from other hospitals. In addition, our study was an observational design, so although the monitor appears to have some predictive value, we do not know if acting on that information would have improved outcome. Unfortunately, there was no established published criteria for a postoperative “respiratory event” we could use. However, our criteria were defined a priori and attempted to capture signs/symptoms and treatments for respiratory events that we believe clinicians would find meaningful. Finally, prior to analysis of the primary endpoint, we excluded all of the data from one of the sites. It appeared that event data were not captured systematically and in real time by a study team member at this site, as was done at the US site. Of note, despite, excluding that site’s data, we retained very good power for the study, since event rates were higher than predicted.

Our study has several strengths. It was prospective and blinded, with all event definitions and analysis methods determined a priori prior to enrollment. In addition, a dedicated study team member stayed continuously at the bedside during the entire data collection period to ensure comprehensive identification and the exact timing of all events. We did not want to rely on potentially inaccurate times for events in the EMR. We also synchronized the clocks for the Capnostream monitor and the study team member’s data recording tool to ensure that there was no discrepancy between these two times. Another strength of the study was our use of exploratory analyses to assess the performance of the monitor in several ways. For example, while we defined early success as the IPI decreasing within 2 to 15 min prior to a respiratory event, we also assessed late success, which extended the time to 2 min before and 2 min after an event occurred. Finally, the outcomes of sensitivity and false discovery are meaningful to the practical utility of the device. It important to identify what proportion of events would be detected by using this monitoring system. It is also important to the utility of the device to understand what proportion of the positive detections were false alarms.

In summary, in our study of 358 patients recovering from general anesthesia, we found that a low IPI was 75.6% sensitive for *early detection* (within 2–15 min *prior* to) of respiratory events but did not achieve our preset threshold of 80% for success.

## Supplementary Information

**Additional file 1: Supplemental Table 1.** Exploratory: All low IPI events for false discovery rates (no washout). **Supplemental Table 2.** Sensitivity and specificity considering only the first occurrence (in time) of either an event or a low IPI value for early or on time success.

## Data Availability

The datasets used and analyzed during the current study are available from the corresponding author on reasonable request as permitted by IRB regulations.

## Abbreviations

RR: Respiratory rate (RR)
FDA: Food and Drug Administration
IPI: Integrated pulmonary index
CI: Confidence interval
EtCO_2_: End-tidal CO_2_
PACU: Post-anesthesia care unit
IRB: Institutional Review Board
BUN: Blood urea nitrogen
COPD: Chronic obstructive pulmonary disease
PRN: Pro re nata
BANG: Body mass index, Age, Neck Circumference, Gender
ICU: Intensive care unit
ECG: Electrocardiogram
EMR: Electronic medical record
SD: Standard deviation
IQ: Interquartile range
