# Supplementary material for: Ability of the integrated pulmonary index to predict impending respiratory events in the early postoperative period
Source: Perioper Med (Lond). 2023 Jul 17;12:39. doi: 10.1186/s13741-023-00322-2 (PMC10351196; doi:10.1186/s13741-023-00322-2)
Supplement: Supplementary file 1 — Additional file 1: Supplemental Table 1. Exploratory: All low IPI events for false discovery rates (no washout). Supplemental Table 2. Sensitivity and specificity considering only the first occurrence (in time) of either an event or a low IPI value for early or on time success. [file 13741_2023_322_MOESM1_ESM.docx]

**SUPPLEMENTAL Table**

**Exploratory: All low IPI Events for False Discovery Rates (no washout)**

*Taking ALL low IPI events (no washout periods), matching with observed events, defined the same as the FP analysis*

| **Low IPI Events Per Epoch: Each patient's IPI data were divided into 4 30-minute epochs. Total number of low IPI events were averaged per epoch. (Minimum 0, Maximum 60 events per epoch)** | | | | | | |
| --- | --- | --- | --- | --- | --- | --- |
|  | **Average** | **SD** | **Median** | **IQR** |  |  |
| Epoch 1 | 10.3 | 13.6 | 4 | 0, 16 |  |  |
| Epoch 2 | 10.1 | 14.7 | 2 | 0, 15 |  |  |
| Epoch 3 | 9.9 | 14.3 | 2 | 0, 16 |  |  |
| Epoch 4 | 8.7 | 14.1 | 2 | 0, 11 |  |  |
| Total (Max 120 Minutes Monitored) | 36.7 | 46.0 | 16 | 2, 57 |  |  |

* Individuals had a median of 16 (IQR 2, 57) IPI events during the 120 minute monitoring period.

IPI=integrated pulmonary index

| **Total Participants with Low IPI Events** | 292 |  |
| --- | --- | --- |
| **Total Low IPI Events (no washouts)** | 13158 |  |
| Total # Early Successful identification of an observed Event | 2462 | 18.7% |
| Total # Partial/On Time successful identification of an observed Event (*no overlap occurred) | 2192 | 16.7% |
| Ontime + Early Success | 4634 | 35.2% |
| **False Discovery Rate (early success only)** | 2462 | 81.3% |
| **False Discovery Rate (early and on time success)** | **4634** | **64.8%** |

*When considering all low IPI events with no washout periods, false positive rates were slightly lower (64.8% vs. 70.5%), but still higher than the target 35%.

There were no adverse events attributable to the monitor.
